# Tic Douloureux, or Neuralgia Facialis, and Other Nervous Affections: Their Seat, Nature, and Cause. With Cases Illustrating Successful Methods of Treatment

**Published:** 1843-10

**Authors:** 


ART. VII.—*Tic Douloureux, or Neuralgia Facialis, and other Nervous Affections: their Seat, Nature, and Cause. With Cases illustrating successful methods of Treatment.* By R. H. ALLNATT, M.D. A.M. F.S.A. SECOND EDITION.—*London*, 1843. 8vo, pp. 224.

WE made a brief note of the character of Dr. Allnatt's book on its first appearance, in October, 1841. (See vol. XII. p. 524.) We notice its reappearance in its present form, chiefly for the purpose of inquiring whether the author is justified in now announcing it as a SECOND EDITION. There has been no *reprint* of the work; this so-called second edition differing only from the former in having a new title-page, containing, in addition, the words "second edition" and the date "1843," in place of "1841," and in having a "superadded sequel to the appendix" of some thirty or forty pages, containing "additional cases successfully treated by the author." Let us put these questions to Dr. Allnatt: Suppose any one of our protegées, Drs. and Messrs. Ramadge, Turnbull, Yearsley, Curtis, &c. &c., was to print *annually* a "superadded sequel of cases successfully treated by the author," tack this to the tail of all the remaining copies of his book, and then announce the cobbled fabric as a NEW EDITION,—could he not plead the same justification as Dr. Allnatt himself? By such a proceeding, would not our respected protegée obtain great advantages, necessarily attaching to the credit and flowing from the reputation of having written so good a book that the public call for a new edition every year? What would Dr. Allnatt think of such conduct? Would he not be apt to set it down as one of the multiform aspects of that worst of all quackery—quackery within the professional sanctuary? And yet would not these learned doctors and surgeons have precisely the same good reason for calling their annual puff a new edition, as Dr. Allnatt has for calling the volume before us a *Second Edition*?

If we did not know Dr. Allnatt to be a gentleman of honorable feelings and intentions, we would not put these questions to him. But we are desirous that dishonorable men should not be able to obtain any sort of sanction from the proceedings of their betters: and this nuisance of spurious New Editions we are determined on abating.

